# Structural insight into glucose repression of the mannitol operon

**DOI:** 10.1038/s41598-019-50249-2

**Published:** 2019-09-26

**Authors:** Mangyu Choe, Huitae Min, Young-Ha Park, Yeon-Ran Kim, Jae-Sung Woo, Yeong-Jae Seok

**Affiliations:** 1 School of Biological Sciences and Institute of Microbiology, Seoul National University, Seoul, 00826 Korea; 2 Department of Life Sciences, Korea University, 145 Anam-ro, Seongbuk-gu, Seoul, 02841 Republic of Korea

**Keywords:** Bacteriology, X-ray crystallography

## Abstract

Carbon catabolite repression is a regulatory mechanism to ensure sequential utilization of carbohydrates and is usually accomplished by repression of genes for the transport and metabolism of less preferred carbon compounds by a more preferred one. Although glucose and mannitol share the general components, enzyme I and HPr, of the phosphoenolpyruvate-dependent phosphotransferase system (PTS) for their transport, glucose represses the transport and metabolism of mannitol in a manner dependent on the mannitol operon repressor MtlR in *Escherichia coli*. In a recent study, we identified the dephosphorylated form of HPr as a regulator determining the glucose preference over mannitol by interacting with and augmenting the repressor activity of MtlR in *E. coli*. Here, we determined the X-ray structure of the MtlR-HPr complex at 3.5 Å resolution to understand how phosphorylation of HPr impedes its interaction with MtlR. The phosphorylation site (His15) of HPr is located close to Glu108 and Glu140 of MtlR and phosphorylation at His15 causes electrostatic repulsion between the two proteins. Based on this structural insight and comparative sequence analyses, we suggest that the determination of the glucose preference over mannitol solely by the MtlR-HPr interaction is conserved within  the *Enterobacteriaceae* family.

## Introduction

Living organisms can use various compounds as carbon sources. Efficient uptake and metabolism of these compounds are crucial for their survival and growth under fluctuating and competitive environmental conditions. One way to obtain higher efficiency is reducing synthesis and/or activity of the peripheral catabolic enzymes if a rapidly metabolizable carbon source is available. This general regulatory phenomenon is called carbon catabolite repression (CCR)^1^. In most heterotrophic bacteria, sugars transported by the phosphoenolpyruvate:sugar phosphotransferase system (PTS) usually suppress synthesis and/or activity of the catabolic enzymes for non-PTS sugars. For example, in *E. coli*, PTS sugars such as glucose and mannitol are preferred to non-PTS sugars such as lactose, glycerol, and maltose^2,3^.

The PTS is a multicomponent and multifunctional system that comprises enzyme I (EI), histidine phosphocarrier protein (HPr), and various sugar-specific enzyme II proteins (EIIs). The phosphoryl group of phosphoenolpyruvate (PEP) is sequentially transferred to EI, to HPr, to various EIIs, and finally to the incoming sugars. Therefore, it concurrently couples transport of sugars to their phosphorylation, using PEP as the energy source^4,5^. Notably, the phosphorylation states of PTS components change depending on the type and availability of sugars. For example, in the presence of a preferred PTS sugar such as glucose, glucose-specific EIIA component (EIIA^Glc^) and HPr are mostly dephosphorylated, whereas their phosphorylated forms increase in the presence of non-PTS sugars in *E. coli*^6–8^. Therefore, the ratio of dephosphorylated to phosphorylated forms can serve as a signal for sensing the sugar availability that leads to regulation of various metabolic pathways including CCR^3,4^.

Preferential utilization of carbon sources (CCR) has been extensively studied in *E. coli*, especially for glucose preference over lactose (glucose-lactose diauxie)^3,9^. It is generally accepted that glucose preference over lactose involves two distinct mechanisms, inducer exclusion and cAMP synthesis inhibition, both of which are strictly dependent on the phosphorylation state of EIIA^Glc ^^3^. First, non-PTS sugar transporters such as lactose permease are inhibited by dephosphorylated EIIA^Glc^ through direct interaction during glucose consumption^8^. This is termed “inducer exclusion”. Second, the activity of adenylate cyclase, which is responsible for the synthesis of cAMP, is activated only by phosphorylated EIIA^Glc ^^7^. Since cAMP induces expression of various genes for the transport and/or metabolism of less preferred carbon sources, secondary carbon sources cannot be utilized when a preferred carbon source such as glucose is available.

Although mechanisms of the preference between glucose and non-PTS sugars have been extensively studied, the regulatory mechanism underlying preference among PTS sugars is still poorly understood. In our recent study on the preference between two PTS sugars, glucose and mannitol, we found that the preference of glucose over mannitol is determined by the phosphorylation state of HPr, a general component commonly used for most PTS sugars including glucose and mannitol, but not by that of EIIA^Glc ^^10^. In the presence of glucose, HPr is mostly dephosphorylated, and the mannitol operon repressor (MtlR) recognizes dephosphorylated HPr to form the HPr-MtlR complex, which serves as a strong repressor of the mannitol operon (Supplementary Fig. 1). In the presence of mannitol alone, however, more than 50% of HPr exists in phosphorylated forms, which cannot interact with MtlR. Using the K27E mutant of HPr, which retains the phosphotransferase activity comparable to that of wild-type HPr but does not interact with MtlR, we could show that the interaction between HPr and MtlR is sufficient to confer the glucose preference over mannitol.

To understand the precise mechanism of the sugar-dependent on/off switching of the *mtl* operon, we first sought to establish how MtlR distinguishes the phosphorylation state of HPr. Here we solved the crystal structure of the *E. coli* MtlR-HPr complex to obtain structural insight into the molecular mechanism of how MtlR mediates the glucose signal. We also investigated how widespread the regulation of the *mtl* operon by the MtlR-HPr complex is among bacteria.

## Results

### *E. coli* MtlR forms a homodimer with an HPr-binding site on each protomer

**Figure 1:**
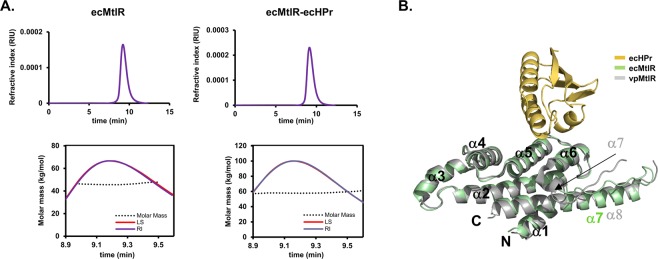
*E. coli* MtlR and HPr forms a stable heterotetramer. (**A**) The SEC-MALS analysis of ecHisMtlR (left) and the ecHisMtlR-HPr complex (right). Refractive index (RI) and light scattering (LS) signals were measured to determine protein concentration (top panels) and molar mass (bottom panels), respectively. (**B**) Structure of the ecMtlR monomer (green) in complex with HPr (yellow). The vpMtlR structure^11^ (PDB 3BRJ, gray) is superposed on the ecMtlR structure. N- and C-termini and α-helices of both MtlRs are labeled. vpMtlR has an additional α-helix (α7 in gray) compared with ecMtlR, thus α8 of vpMtlR in gray and α7 of ecMtlR in green are structurally aligned.

*Vibrio parahaemolyticus* MtlR (vpMtlR), a close homolog of *E. coli* MtlR (ecMtlR), has been previously reported to form a stable homodimer through extensive hydrophobic interactions^11^. Since the residues lining the dimeric interface of MtlR are highly conserved between the two species, it was likely that ecMtlR also forms a homodimer. To verify this, we performed size exclusion chromatography (SEC) followed by multi-angle light scattering analysis (MALS) with purified ecMtlR fused with His_6_-tag (ecHisMtlR: ~23 kDa) (Fig. 1A). The SEC result showed a single monodisperse peak, and the molecular mass estimated from the MALS data was ~45 kDa, indicating that ecHisMtlR exists as a homodimer. Next, we purified the ecHisMtlR-HPr complex and measured its molecular mass in SEC-MALS. The resulting molecular mass of the complex was ~59 kDa, which is slightly smaller than the theoretical mass (~64 kDa) of the 2:2 complex between ecHisMtlR and HPr, suggesting that one or two molecules of HPr (9 kDa) are bound to the MtlR dimer.

The purified complex was initially crystallized in the reservoir solution containing 100 mM Tris-HCl (pH 8.5), 200 mM Mg-acetate, and 15% polyethylene glycol (PEG) 8000, and yet the crystals were very small and did not diffract X-rays to sufficient resolution for three-dimensional structure analysis. While changes in the reservoir condition could not improve the crystal quality, the addition of purified HPr into the protein complex sample dramatically increased the size and diffraction quality of crystals. From an improved crystal, we collected the 3.5 Å resolution data and solved the ecMtlR-HPr complex structure by the molecular replacement method using the available vpMtlR structure and an *E. coli* HPr structure (see Methods). In the crystal structure, the asymmetric unit contains two ecMtlR monomers, which do not form an apparent dimer with each other. Instead, each ecMtlR monomer forms a dimer with a symmetry-related molecule. As expected from the sequence homology with vpMtlR, the dimeric interaction of ecMtlR was almost identical to that of vpMtlR. Importantly, we identified that an HPr molecule is bound to each of the two ecMtlR monomers in the asymmetric unit. The two ecMtlR-HPr complexes in different crystal packing environments exhibited almost identical structures (data not shown). The structural alignment of HPr-bound ecMtlR with vpMtlR also showed no significant conformational difference between the two MtlRs, suggesting that HPr-binding does not change the overall structure of ecMtlR (Fig. 1B). Therefore, it is most likely that the two ecMtlR protomers in the dimer interact with HPr independently of each other. Hence the ecMtlR-HPr complex can be in 2:1 or 2:2 stoichiometry of ecMtlR and HPr depending on the concentration of dephosphorylated HPr.

### Overall structure of the heterotetrameric ecMtlR-HPr complex

**Figure 2:**
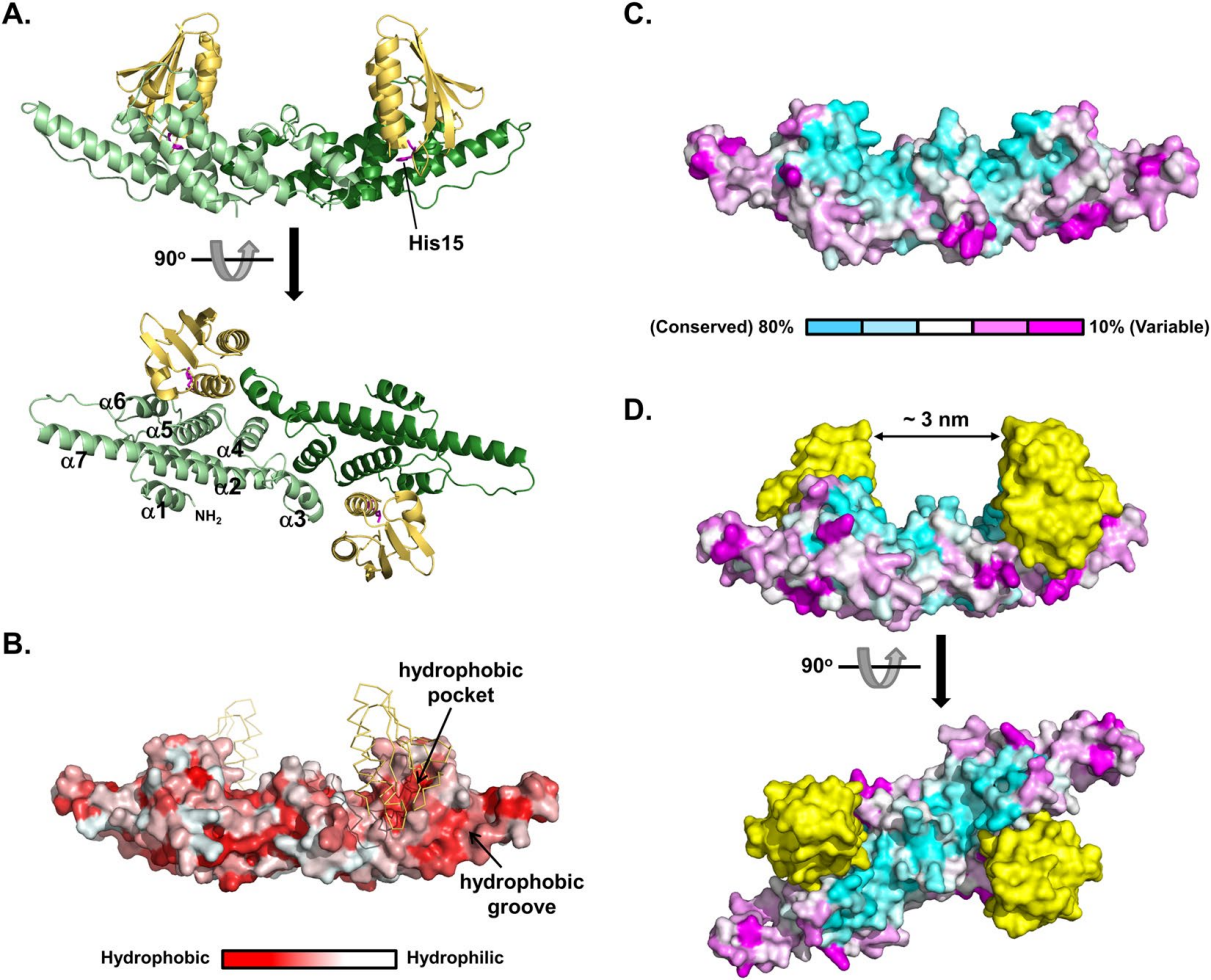
Overall structure of the MtlR-HPr complex and surface-properties of the MtlR dimer. (**A**) Ribbon diagrams showing two orthogonal views of the MtlR-HPr complex. The MtlR-HPr complex is a heterotetramer composed of two MtlR and two HPr molecules. MtlR and HPr are shown in green and yellow, respectively. α-Helices of MtlR are labeled and His15 of HPr is shown in a magenta stick. (**B**) Hydrophobicity of the surface amino acids in the MtlR dimer. The ecMtlR dimer was colored according to the Eisenberg hydrophobicity scale^36^ and the “Color h” script in the Pymol program. (**C**) Conservation of the surface amino acids in the MtlR dimer. The conservation rate of each residue of MtlR was calculated by multiple sequence alignment as described in the Materials and Methods section. Twenty-one non-*Enterobacteriaceae* MtlR sequences were aligned and shown as surface presentation. The color bar indicates the conservation rate. (**D**) The conserved w-shaped groove formed by the binding of HPr (yellow) to MtlR.

Rather than the predicted α/β structure^12^, the ecMtlR monomer consists exclusively of α-helices arranged in 3 layers (α1 in the first layer; α2, α3, and α7 in the second; α4, α5, and α6 in the third), which superpose well with the corresponding helices of vpMtlR (Fig. 1B). However, the shortest helix (α7) of vpMtlR is replaced by a loop in ecMtlR, and the conformation of the proline-rich loop region (residues 145–157) between α6 and α7 in ecMtlR is quite different from that of the corresponding loop in vpMtlR (Fig. 1B). Notably, 10 out of 13 residues in this loop of ecMtlR are hydrophobic and mostly solvent-exposed, and thus a wide hydrophobic groove is formed by the α6-α7 loop together with helices α6 and α7. The groove-lining residues are quite well conserved in the *Enterobacteriaceae* family. EcMtlR homo-dimerizes mainly through interactions mediated by the helices α3 and α4, resulting in an elongated ship-shaped structure (Fig. 2A). Since the dimeric interaction is almost identical to that of vpMtlR, the overall structure of the dimer is also similar between the two proteins. From the amino acid sequence alignment of 21 MtlR homologs selected following a BLAST search for sequences sharing 20–50% identity with *E. coli* MtlR and also with one another, we found that a broad surface area corresponding to the upper deck of the ship-shaped structure is highly conserved (Fig. 2C), suggesting that this area may be involved in the interaction with an unknown binding partner such as a cognate DNA-binding protein proposed in our previous study^10^. Interestingly, HPr bound to the hydrophobic pocket formed by α5 and α6 between the hydrophobic groove and the highly conserved area (Fig. 2B,C). Consequently, two HPr molecules on the MtlR dimer are facing each other across the conserved area forming a deep groove, which is enough to accommodate a small protein domain or double-stranded DNA (Fig. 2D).

### Hydrophobic interaction is crucial for the ecMtlR-HPr binding

**Figure 3:**
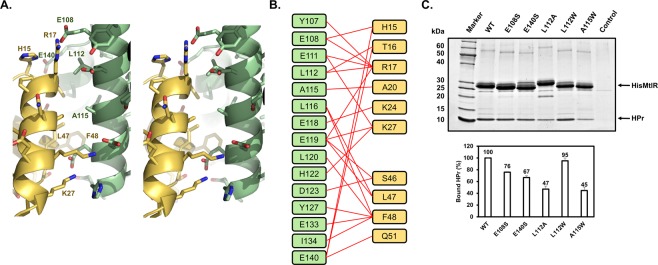
The Binding interface between MtlR and HPr. (**A**) Stereoview of the MtlR-HPr interface. Proteins are color-coded as in Fig. 2. Amino acid side chains are shown in sticks. The amino acids subjected to the mutational study are labeled. (**B**) Schematic diagram denoting the amino acid interactions between MtlR and HPr. Red lines indicate the amino acid pairs in which the interatomic distance is less than 5 Å. (**C**) *In-vitro* binding assays of MtlR variants with HPr. Wild-type or mutant His-MtlR (200 μg each) was mixed with HPr (200 μg) in the binding buffer containing 20 mM Tris-HCl (pH 7.5), 50 mM NaCl, 2 mM β-mercaptoethanol and 5% glycerol and then subjected to Talon metal affinity chromatography (Clontech Laboratories, Inc.). After 3 washes with the binding buffer with 10 mM imidazole, the bound proteins were eluted with the binding buffer containing 150 mM imidazole. Eluted samples were analyzed by 4–20% SDS-PAGE followed by staining with Coomassie brilliant blue R-250. Representative data from three independent experiments are shown. The band intensities of bound HPr were analyzed using Multi Gauge version 3.0 software and normalized by the band intensities of each MtlR. The amount of HPr bound to each mutant MtlR was then expressed as a percentage of that bound to wild-type MtlR and shown below each lane.

The binding interface between ecMtlR and HPr is formed by 15 residues from ecMtlR and 10 residues from HPr, where the interatomic distance is less than 5 Å (Fig. 3A,B). The most prominent interaction in the interface is between Phe48 in HPr and the hydrophobic pocket of the ecMtlR surface (Supplementary Fig. 2). Similar interactions have also been observed in the structures of Rsd-HPr and EIIA^Mtl^-HPr complexes, suggesting that Phe48 of HPr is widely used for its recognition by other proteins^13,14^. In addition to Phe48, its neighboring hydrophobic residue Leu47 closely interacts with the top rim of the pocket. The importance of the two residues for the ecMtlR binding was evidenced by our previous mutational study, in which the double mutation of L47A and F48A in HPr significantly decreased the binding affinity for ecMtlR^10^.

To further verify whether the ecMtlR-HPr structure represents the interaction of the two proteins in solution, we also mutated two hydrophobic residues in the binding surface on MtlR (Fig. 3C). We first substituted Ala115 with tryptophan. This alanine residue is completely solvent-exposed when HPr is not bound, and interacts with Ala20 of HPr upon its binding. Hence we assumed that a mutation of Ala115 of MtlR to a larger residue may cause a steric hindrance with Ala20 of HPr without affecting its structure. The A115W mutant of His-tagged ecMtlR was expressed in a soluble form in *E. coli*, and we purified the mutant as well as the wild-type protein for the His-tag pull down assay. We mixed His-MtlR or its mutant protein (200 μg each) with 200 μg of HPr, and loaded the mixture onto a TALON metal affinity column. After a brief wash, proteins bound to the TALON resin were eluted with 150 mM imidazole and analyzed by SDS-PAGE followed by staining with Coomassie brilliant blue. On the stained gel, the band intensity of HPr co-eluted with His-MtlR(A115W) was less than half of that co-eluted with the wild-type His-MtlR, indicating that the A115W mutation significantly decreased the binding affinity for HPr. We also mutated Leu112, which is half-buried in the binding surface of MtlR and interacts with Thr16 and Arg17 of HPr. When Leu112 was mutated to Trp, there was no apparent decrease in the HPr-binding affinity. However, when Leu112 was mutated to Ala, the HPr-binding activity was significantly decreased, indicating a bulky hydrophobic residue is required at this position.

### The electrostatic interaction is crucial for the ecMtlR-HPr binding and can be compromised by phosphorylation at His15 of HPr

**Figure 4:**
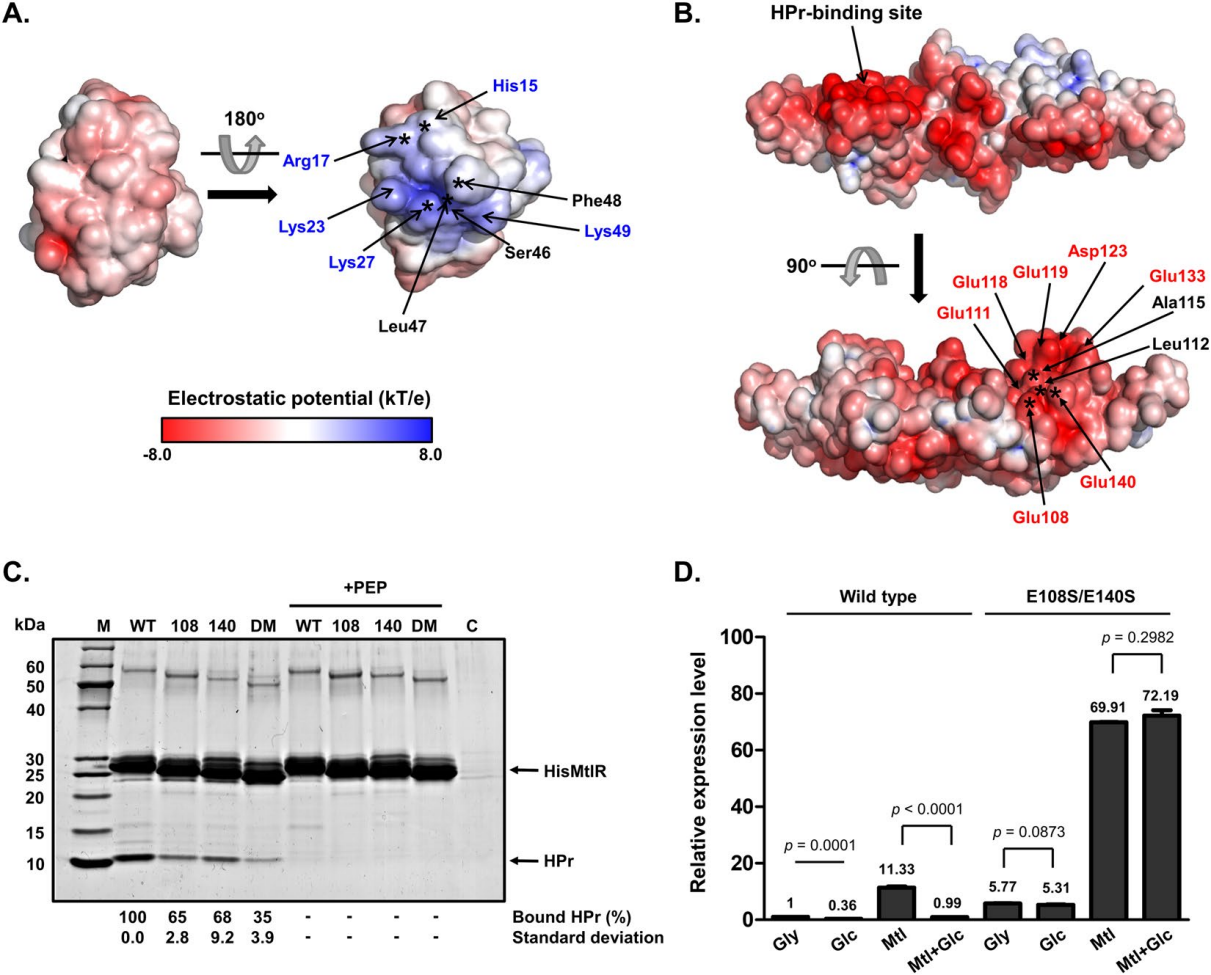
The MtlR-HPr interaction is the key mechanism for the glucose repression of the *mtl* operon in *E. coli*, which is independent of the mannitol induction mechanism. (**A**,**B**) Surface electrostatic potential of HPr and MtlR. The Adaptive Poisson-Boltzmann Solver (APBS) was used to calculate the electrostatic potential at pH 7. The potential ranging from −8 kT/e to 8 kT/e is colored as indicated by the color spectrum bar at the bottom in A. The potential lower than −8 kT/e is colored in red, and that higher than 8 kT/e is colored in blue. The interaction surface of HPr (**A**) contains several basic residues (indicated in blue) whereas that of MtlR (**B**) has several acidic residues (indicated in red). The residues identified to be involved in complex formation in previous and this study are indicated by asterisks on the surfaces of HPr and MtlR, respectively. (**C**) Tests of interaction between MtlR variants and HPr. Wild-type, E108S, E140S, or E108/140S double mutant (DM) of His-MtlR (200 μg) was mixed with 20 μl of EI-overproducing cell lysate, 200 μg of HPr, 2 mM MgCl_2_ and 50 μl of Talon metal affinity resin (Clontech Laboratories, Inc.) in the absence or presence of 2 mM PEP as indicated. Each mixture was then subjected to a pull-down assay and processed as described in the legend to Fig. 3C. Experiments were repeated three times with reproducible results. Representative data from three independent experiments are shown. The band intensities of HPr were analyzed as described in the legend to Fig. 3C. (**D**) Effect of the *mtlR*(E108S/E140S) mutation on expression of the *mtl* operon in the presence of different sugars. The wild-type *E. coli* MG1655 and the chromosomal *mtlR*(E108S/E140S) mutant were grown in M9 medium containing 0.2% of indicated sugar(s) (Gly, glycerol; Glc, glucose; Mtl, mannitol; Mtl/Glc, glucose and mannitol). Cells were harvested at mid-exponential phase and the expression level of *mtlA* was quantified by qRT-PCR. Means and standard deviations from three independent experiments are shown relative to that in glycerol-grown wild-type cells, and statistical significance (*p* value) was determined by Student’s *t*-test.

An interesting feature of the intermolecular interaction is the charge distribution in the interface. We found that the binding surface of HPr contains five basic residues and no acidic residue, while that of ecMtlR has seven acidic residues (Glu108, Glu111, Glu118, Glu119, Asp123, Glu133, and Glu140) and only one basic residue (His122) (Fig. 3B). As shown in the electrostatic potential surfaces (Fig. 4A,B), several basic residues (marked by blue letters) of HPr are clustered near the phosphorylation site (His15), while several acidic residues (marked by red letters) of MtlR are clustered around the HPr-binding site. This charge distribution suggests that the electrostatic interaction may be the main contributor to the binding affinity of the two proteins. In the previous study, we showed that the binding affinity for MtlR significantly decreased in several HPr mutants, including R17A, K27E, and S46D^10^. Therefore, the introduction of a negative charge(s) and/or removal of a positive charge(s) near the phosphorylation site of HPr appear to have influenced the MtlR-HPr interaction. In the MtlR-HPr structure, Arg17 and Lys27 of HPr can form salt bridges with Glu111 and Glu119 of MtlR, while Ser46 of HPr is located close to Glu119 and Asp123 of MtlR (Fig. 3B), providing mechanistic explanations for the observed decreases in the binding affinity of those HPr mutants for MtlR^10^.

To further verify the importance of the electrostatic interaction, we also mutated two glutamates on the binding surface of ecMtlR and performed an *in vitro* binding assay (Figs 3C and 4C). As expected, E108S and E140S mutants showed decreased binding affinities for HPr. We also tested a double mutant at both residues, and the mutant showed a much lower affinity for HPr than the single mutants. Although how the glutamate residues interact with HPr is not clear due to the low resolution of the current structure, we assume that Glu108 and Glu140 interact with basic residues near the His15 phosphorylation site. To determine precise binding affinities of HPr for the wild-type and mutant ecMtlR, we performed interaction tests using Monolith NT.115 (NanoTemper Technologies) (Supplementary Fig. 3). The measured dissociation constant (K_D_) for the wild-type complex was ~21 nM, which is slightly lower than the data previously determined by Surface Plasmon Resonance (SPR)^10^. As expected, the K_D_ values for E108S and E140S single mutants were 1.42 ± 0.08 μM and 1.53 ± 0.11 μM which are significantly increased (~68 and ~73 fold, respectively) and the K_D_ value for the double mutant was too high to measure by this method. These data strongly suggest that the electrostatic interaction is the major determinant of the ecMtlR-HPr interaction.

Given the importance of the surface charge for the ecMtlR-HPr interaction, the introduction of negative charges due to phosphorylation at His15 of HPr is likely to cause charge repulsion with Glu108 and/or Glu140 of MtlR and therefore prevent the interaction between the two proteins. Together, the localization of His15 of HPr at the binding interface for MtlR explains why phosphorylation at His15 abolishes its interaction with MtlR.

### The MtlR-HPr interaction is the key mechanism for the glucose repression of the *mtl* operon in *E. coli*, which is independent of the mannitol induction mechanism

We previously showed that K27E mutant of HPr still retains the phosphotransferase activity but is unable to interact with ecMtlR, which led to a loss of glucose preference over mannitol^10^. However, since HPr is one of the most highly conserved proteins in bacteria and interacts with many other proteins involved in several physiological processes including Rsd, glycogen phosphorylase and the transcriptional antiterminator BglG in a phosphorylation-dependent manner^15–18^, we could not completely exclude the possibility that the effect of the K27E mutation in HPr was due in part to a loss of the interaction with its binding partners other than MtlR. Since the introduction of mutations at both Glu108 and Glu140 in ecMtlR almost completely abolished the HPr binding, we used this double mutation to confirm whether the interaction between MtlR and HPr is sufficient to determine the glucose repression of the *mtl* operon in *E. coli*.

We introduced the E108S and E140S mutations into the *mtlR* gene of the *E. coli* chromosome. The wild-type and mutant strains of *E. coli* were grown in M9 minimal medium containing glycerol, mannitol, or glucose and the mRNA levels of *mtlA* encoding the mannitol-specific PTS permease were measured. In the wild-type *E. coli* cells grown on glycerol, ~38% of HPr is known to exist in a dephosphorylated form^10^. Therefore, it could be assumed that at least a small amount of MtlR is in the HPr-bound state and thus the *mtl* operon would be repressed to some extent even in the presence of glycerol alone. In accordance with this assumption, the *mtlA* transcript level was ~6 fold higher in the chromosomal *mtlR*(E108S/E140S) mutant than in the wild-type strain (Fig. 4D, lanes 1 and 5). In the presence of glucose, however, ~88% of HPr is dephosphorylated^10^, and this resulted in significantly stronger repression of the *mtl* operon than in wild-type cells grown on glycerol (Fig. 4D, lanes 1 and 2). However, in the mutant strain, expression of the *mtl* operon was not subject to significant repression by glucose regardless of the presence of mannitol (Fig. 4D, lanes 5 to 8), indicating that the glucose repression is not functioning in the mutant strain. These data support our previous conclusion that the MtlR-HPr interaction is the key mechanism for the glucose repression of the *mtl* operon in *E. coli*^10^.

In our previous study^10^, we showed that expression of the *mtl* operon is not fully induced in the presence of both glucose and mannitol when compared to that observed in the presence of mannitol alone in wild-type *E. coli*. We therefore concluded that the MtlR-HPr interaction inhibits the derepression of the *mtl* operon by mannitol. However, we could not clarify whether dephosphorylated HPr blocks the derepression of the *mtl* operon by directly inhibiting the inductive effect of mannitol or whether the mechanisms for glucose repression and mannitol induction occur independently of each other. In the wild-type strain, the *mtlA* mRNA level increased ~11 folds by mannitol in the medium, compared to glycerol medium (Fig. 4D, lanes 1 and 3). Interestingly, a similar degree of mannitol induction of *mtlA* expression (~12 fold) was observed in the mutant strain (Fig. 4D, lanes 5–8), indicating that the *mtlR*(E108S/E140S) mutant has lost the repressibility by glucose but still retains its inducibility by mannitol. Therefore, these data suggest that the MtlR-HPr interaction is independent of the mechanism for mannitol induction of the *mtl* operon. However, further studies are required to elucidate how MtlR binds to the promoter region and how mannitol interferes with the repression of the *mtl* operon by MtlR.

### The MtlR-HPr interaction is conserved within the *Enterobacteriaceae* family

**Figure 5:**
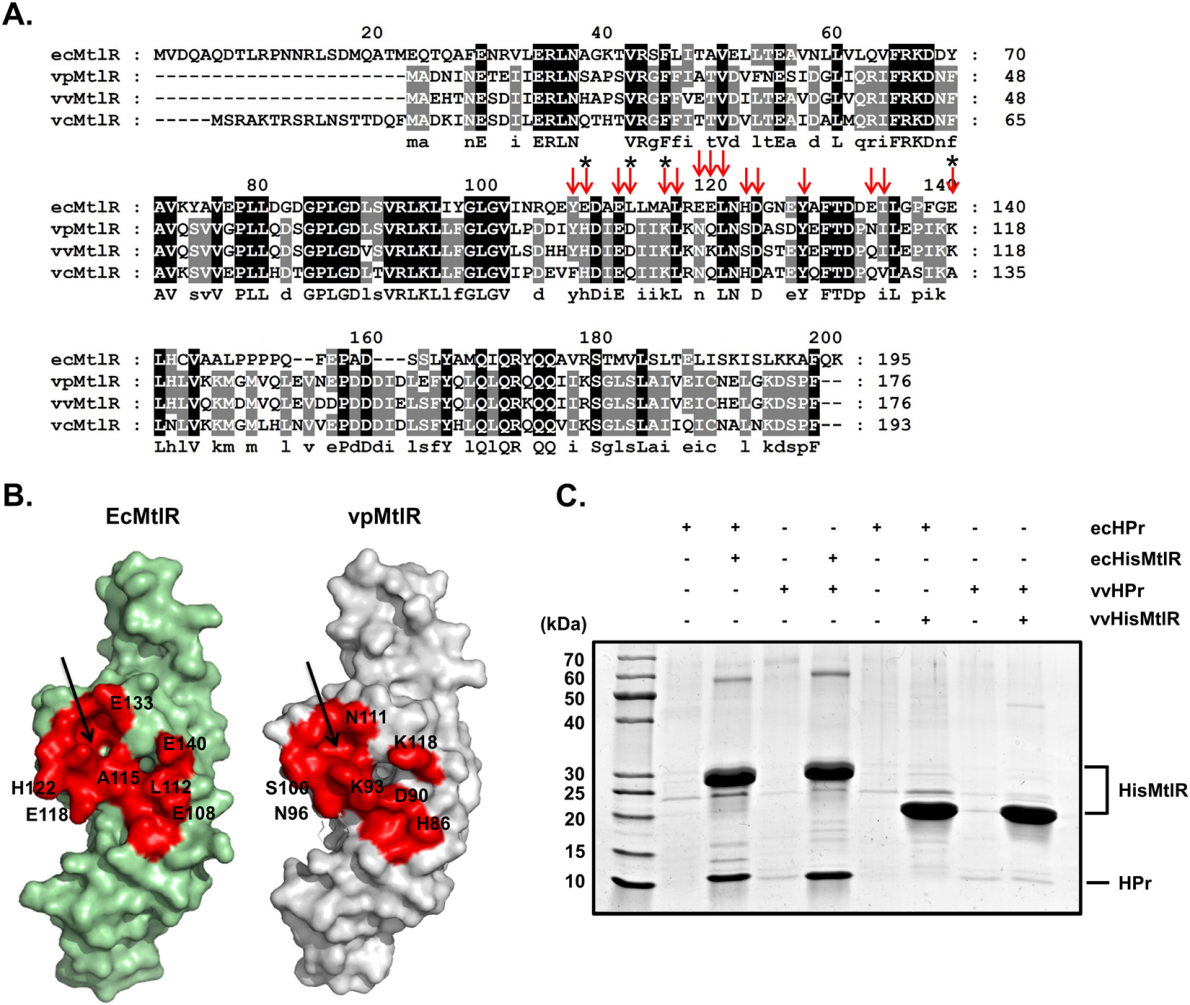
The MtlR-HPr interaction is conserved within the *Enterobacteriaceae* family. (**A**) Amino acid sequence alignment of MtlRs among *E. coli* and *Vibrio* species using ClustalX2. The conserved residues are highlighted in black (100%) and gray (75%). The residues involved in the interaction with HPr are indicated by red arrows. Four residues tested in Fig. 3C are indicated by asterisks. ec, *E. coli*; vp, *V. parahaemolyticus*; vv, *V. vulnificus*; vc, *V. cholerae*. **(B**) Comparison of the HPr-binding surface on ecMtlR with the corresponding surface on vpMtlR (colored in red). The residues that are not identical between ecMtlR and vpMtlR are labeled with numbers on their surfaces. The conserved hydrophobic pockets are indicated by arrows. (**C**) Tests of interaction between MtlRs and HPrs. Both *E. coli* and *V. vulnificus* proteins (HisMtlR and HPr) were used for the binding tests. His-tagged MtlR (200 μg) from *E. coli* or *V. vulnificus* was mixed with HPr (200 μg) from *E. coli* or *V. vulnificus* and subjected to Talon metal affinity chromatography. After a three-time wash, bound proteins were eluted and analyzed by 4–20% SDS-PAGE and staining with Coomassie brilliant blue.

Since the phenomenon of carbon catabolite repression is observed in most bacteria including *Vibrio* species^3,19^ and the structural alignment of HPr-bound ecMtlR with vpMtlR showed no significant conformational difference between the two MtlRs (Fig. 1B), we reasoned that the mechanism for glucose repression of the *mtl* operon might be conserved in species belonging to the *Vibrionaceae* family. Interestingly, however, Glu108 and Glu140 in ecMtlR are not conserved in *Vibrio* MtlRs at all, although the Phe48-binding pocket is quite well conserved in both *Vibrio* and *E. coli* species (Fig. 5A,B). This observation led us to assess whether *Vibrio* MtlRs also interact with dephosphorylated HPr. We purified HPr and MtlR from *V. vulnificus* (vvHPr and vvMtlR) and *E. coli* (ecHPr and ecMtlR) and performed an *in vitro* binding assay and gel filtration chromatography (Fig. 5C and Supplementary Fig. 4). Surprisingly, vvMtlR did not interact with HPrs from either of the two species, whereas ecMtlR bound to vvHPr as tight as to ecHPr. As all the residues lining the MtlR-binding site of HPr (marked with red arrows in Supplementary Fig. 5) are completely conserved among *E. coli* and *Vibrio* species, the variation of the MtlR-HPr interaction between these species is likely due to the variation in the amino acid sequence of MtlR.

Since the two bacterial species showed a clear difference in the MtlR-HPr interaction, we were curious whether this interaction is conserved in other bacterial species. Thus, we performed sequence alignment of MtlRs from ~1000 γ-proteobacterial genomes, and found that Glu108 and Glu140 of ecMtlR are conserved only among *Enterobacteriaceae* MtlRs. Further, we collected MtlR sequences from ~20,000 *Enterobacteriaceae* genome sequences to see if the HPr-binding site is conserved in *Enterobacteriaceae* MtlRs. We found that all the residues lining the HPr-binding site are highly conserved, and especially the acidic residues Glu108, Glu111, Glu118, Glu119, Asp123, Glu133 and Glu140 are almost completely conserved (Supplementary Fig. 6). This finding suggests that MtlRs may share the same HPr binding mechanism in the *Enterobacteriaceae* family and the MtlR-HPr interaction might be the key mechanism for glucose repression of the *mtl* operon in this family.

## Discussion

In this study, we solved a crystal structure of the MtlR-HPr complex and identified that the phosphorylation site His15 of HPr is located close to Glu108 and Glu140 of MtlR. The structure shows that the phosphorylation of HPr introduces negative charges and thereby causes electrostatic repulsion between HPr and MtlR. The introduction of mutations at both Glu108 and Glu140 in MtlR abolished the HPr binding, and expression of the *mtl* operon was not subject to repression by glucose in a chromosomal *mtlR*(E108S/E140S) mutant (Fig. 4C,D). Taken together, our data suggest that glucose repression is entirely dependent on the interaction between MtlR and HPr.

Analysis of the effect of mutations in the charged residues on the binding surface of MtlR and HPr showed that the electrostatic interaction is crucial for their high affinity binding (K_D_ of ~21 nM). In protein-protein interactions, charged residues often contribute to binding specificity rather than binding energy^20,21^. In the case that the charged residues are clustered in the binding surface, it can promote high affinity binding. Clustering of charged residues also facilitates “electrostatic steering,” a long-range mechanism, in which a ligand is driven to its receptor by electrostatic forces. This largely increases the association rate. Although the resolution of the MtlR-HPr structure is not high enough to clearly show how charged residues precisely interact with one another, we identified possible electrostatic interaction pairs (Supplementary Fig. 7), which contribute to the binding specificity. The steering mechanism could also be involved in the MtlR-HPr interaction, since the interaction is mediated by the most negatively charged region of MtlR and the only positively charged area of HPr^22^. Hence, the phosphorylation of His15 of HPr, which is located on the positively charged surface, might disturb the steering of HPr into MtlR and thereby inhibit the specific interaction of Arg17 and His15 with the glutamate residues in MtlR.

Although ecMtlR has a strong binding affinity to HPr, the SEC-MALS data showed that the estimated molecular weight of the complex (~59 kDa) is smaller than expected (~64 kDa), suggesting that the MtlR-HPr mixture might contain a substantial proportion of HPr-free MtlR. This could be caused by two reasons. First, since the injected sample is diluted during size exclusion chromatography, HPr would partially dissociate from MtlR. In agreement with this interpretation, crystallization of the MtlR-HPr complex purified by size exclusion chromatography has been significantly improved by adding purified HPr to the sample. The HPr supplementation likely increased the ratio of the 2:2 complex to the 2:1 complex between MtlR and HPr. Second, although the error of the SEC-MALS experiments with BSA using absorbance at 280 nm was always less than 2%, the experiments with the MtlR-HPr complex could have a larger error because we used the refractive index to measure the protein concentration during SEC-MALS due to the low absorbances of *E. coli* MtlR and HPr at 280 nm. Since the refractive index increments (dn/dc) for these proteins are not known, we used the dn/dc value of 0.185 ml/g, which is widely used for proteins. Given that the dn/dc values for most unmodified proteins range from 0.18 to 0.195^23^, the calculated protein concentration might have ~5% error at most.

HPr is a highly conserved general PTS component that transfers a phosphoryl group from EI to sugar-specific EIIs. HPr has been previously reported to interact with other PTS components and several regulatory proteins, and its structures in complexes with EI^24^, Rsd^14^, LsrK^25^, and four different EIIA proteins^13,26–28^ have been solved so far. These HPr-binding proteins can be categorized into two groups according to the HPr-recognition mechanism. The first group including EI, Rsd, LsrK, and MtlR has three common features. (1) They have a prominent hydrophobic pocket to interact with Phe48 of HPr (Supplementary Fig. 8, upper panels). (2) Several acidic residues on the HPr-binding interface directly interact with the basic surface of HPr. (3) One or two acidic residues are located close to His15 to inhibit the binding of phosphorylated HPr by charge repulsion. In contrast, four EIIA proteins in the second group, which are supposed to recognize phosphorylated HPr, apparently share no common mechanism of the interaction with unphosphorylated HPr (Supplementary Fig. 8, lower panels). It seems that EIIA proteins avoid strong hydrophobic or electrostatic interactions to maintain low affinity for unphophorylated HPr, which may be necessary for their efficient phosphotransfer acitivity.

Dephosphorylated HPr is known to be also recognized by Rsd in *E. coli* to inhibit the expression of stationary-phase genes and stress-responsive genes^14^. In the structure of the Rsd-HPr complex, Rsd also binds to the same basic surface of HPr as MtlR does, and the binding interface of Rsd has five acidic residues, indicating that the electrostatic interaction is also important for the Rsd-HPr interaction. The substitution of the Glu51 residue in Rsd into arginine decreased the binding affinity for HPr ~100 fold^16^, supporting the importance of the electrostatic interaction. Unlike the MtlR-HPr interaction, however, both a steric hindrance and charge repulsion were suggested to be the main reasons why Rsd does not bind to phosphorylated HPr, since His15 of HPr is located very close to the neighboring acidic residues of Rsd (Glu51 and Asp55) upon their binding.

The preferential utilization of glucose over mannitol by bacteria requires precise regulation of the *mtl* operon, in which MtlR plays an important role as a transcriptional repressor^10–12^. In our previous study, the increased ratio of dephosphorylated HPr to phosphorylated HPr was found to be the signal of the glucose presence that is recognized by MtlR^10^. The current study strongly suggests that this recognition is critical for glucose repression of the *mtl* operon in *E. coli*. According to previous studies, the *mtl* operon is likely controlled by at least three regulatory mechanisms: 1) the HPr-dependent repression, 2) an unknown induction mechanism that directly responds to the mannitol concentration, and 3) the cAMP-dependent activation. According to our data in this study, however, the HPr-dependent repression appears to be independent of the induction mechanism by mannitol, since the induction fold of the *mtl* operon by mannitol was similar in the wild type and the *mtlR*(E108S/E140S) mutant (Fig. 4D). In the presence of both glucose and mannitol, however, repression by glucose seems to predominate over induction by mannitol, since the expression of the *mtl* operon is not induced until glucose is exhausted^10^. While the cAMP level decreases in the presence of glucose^7^, glucose did not significantly change the *mtlA* expression level in the *mtlR*(E108S/E140S) mutant (Fig. 4D, lanes 5 and 6). Therefore, the cAMP-dependent activation appears to play, if any, only a minor role in the glucose repression of the *mtl* operon, which is consistent with our previous observation that the addition of cAMP did not affect glucose preference over mannitol of the wild-type *E. coli* strain^10^.

MtlR itself has no DNA binding activity *in vitro* and no DNA binding domain has been found to date^10–12^. However, we previously showed that MtlR specifically binds to the promoter region of the *mtl* operon *in vivo*, although MtlR did not bind to DNA even in the presence of HPr *in vitro*. Therefore, we assumed the existence of a cognate DNA-binding partner of MtlR^10^. Consistently, we could not find any positively charged surface in the MtlR-HPr structure that could strongly bind to DNA. However, since the most negatively charged surface of MtlR is covered by HPr, the MtlR-HPr complex would experience less repulsion from DNA than does MtlR alone. Interestingly, the binding of two HPr molecules to a MtlR dimer forms a symmetrical w-shaped groove with 3 nm width which seems enough to accommodate a small protein domain of a DNA-binding protein (Fig. 2D). Since this groove is lined by highly conserved residues of both MtlR and HPr, it seems to have an important role in the repression mechanism.

## Materials and Methods

### Bacterial strains and plasmids

The bacterial strains and plasmids used in this study are listed in Supplementary Table 1. Bacterial cells were grown as previously described^10,15^. All plasmids used in this study were constructed using standard polymerase chain reaction (PCR)-based cloning procedures and verified by sequencing. Genomic DNA of *E. coli* MG1655, a wild-type K-12 strain, was used as the template DNA for cloning. *E. coli* ER2566 (NEB) was used for overproduction of recombinant proteins. Expression vectors for mutant forms of MtlR were constructed by quick change site-directed mutagenesis using pET-HisMtlR^10^ as the template. In-frame deletion mutants were constructed using the pKD46 plasmid as previously described^29^.

### Media and cell culture conditions

LB medium (1% tryptone, 0.5% NaCl and 0.5% yeast extracts) and TB medium (1.2% tryptone, 2.4% yeast extracts and 0.5% glycerol) were used for routine *E. coli* culture. LBS medium (1% tryptone, 2% NaCl and 0.5% yeast extracts) was used for *V. vulnificus* culture. Ampicillin (100 μg/ml) or kanamycin (20 μg/ml) was added when required. For overproduction of proteins using ER2566 and pET-based expression vectors, cells were grown in LB or TB medium at 37 °C and IPTG was added to the culture medium to a final concentration of 1 mM when the culture reached A_600_ of 0.4 or 1.0, respectively, and the cells were harvested 3–4 h after induction.

### Protein purification

*E. coli* ER2566 transformed with pET-based expression vectors were used for overproduction of His_6_-tagged proteins. His-tagged proteins were purified using Clontech Talon metal affinity resin (Clontech Laboratories, Inc.) according to the manufacturer’s instructions. The cell pellet containing overexpressed proteins were resuspended in binding buffer (20 mM HEPES-NaOH, pH 7.4, 100 mM NaCl, 0.05% β-mercaptoethanol, 10% glycerol), disrupted by two passages through a French pressure cell at 10,000 psi. After centrifugation at 9,300 × g for 20 min to remove cell debris, the soluble fraction was mixed with Talon^TM^ metal affinity resin, and the mixture was loaded onto Poly-Prep chromatography column (8 × 40 mm; Bio-Rad). The column was washed three times with wash buffer (10 mM imidazole added to binding buffer) and the bound proteins were eluted with elution buffer (150 mM imidazole added to binding buffer). The fractions containing His-tagged proteins were concentrated using Amicon Ultracel-3K centrifugal filters (Millipore Ireland). To remove imidazole and other impurities, the concentrated proteins were chromatographed on a Superose 12 10/300 GL column (GE Healthcare Life Sciences) equilibrated with binding buffer.

Untagged proteins were purified using MonoQ^TM^ 10/100 GL and a HiLoad 16/60 Superdex 75 prepgrade columns (GE Healthcare Life Sciences). Cells induced to overexpress HPr or EI were resuspended in buffer A (20 mM Tris-HCl, pH 8.0, 50 mM NaCl, 0.05% β-mercaptoethanol, 5% glycerol) and disrupted by two passages through the French pressure cell at 10,000 psi. After centrifugation at 100,000 × g for 60 min at 4 °C, the supernatant was applied to a MonoQ^TM^ 10/100 GL column equilibrated with buffer A. Protein elution was carried out by using a 15-column volume gradient of 50–1000 mM NaCl in buffer A at a flow rate of 1 ml/min. The fractions containing HPr or EI were pooled, concentrated and chromatographed on a HiLoad 16/60 Superdex 75 prepgrade column equilibrated with the binding buffer.

To purify the MtlR-HPr complex, His_6_-tag and HRV 3C protease cleavage site were fused to the N terminus of the MtlR coding sequence in pET-MtlR. *E. coli* ER2566 harboring this plasmid was grown in LB medium at 37 °C to OD_600_ ~ 0.5 and overexpression of MtlR was induced by 1 mM IPTG for 4 h. Overexpression of HPr was performed using the *E. coli* GI698 strain harboring pSP100 as described previously^18^. The two strains with overexpressed MtlR and HPr were mixed and resuspended in buffer B (20 mM Tris-HCl, pH 7.5, 150 mM NaCl, 2 mM β-mercaptoethanol). After cells were lysed by sonication, the cell lysate was loaded on a Ni-NTA column. The column was washed with buffer B containing 20 mM imidazole and eluted with buffer B containing 200 mM imidazole. The eluate from Ni-NTA was treated with His_10_-tagged HRV 3C protease at 4 °C overnight. The protease-treated sample was diluted 20-fold with buffer B and loaded on a Ni-NTA column to remove the His tag and the protease. The unbound fraction with buffer B was collected and concentrated in a 3 kDa MWCO centricon (Millipore). The concentrated unbound fraction containing the MtlR-HPr complex was then chromatographed on a HiLoad 16/60 Superdex 75 prepgrade column (GE Healthcare Life Sciences) equilibrated with binding buffer. The sample was concentrated up to >40 mg/ml for crystallization screening, LN_2_-cooled and stored at −80 °C.

### Protein crystallization, data collection, and structure refinement

The complex was initially crystallized in the reservoir solution containing 100 mM Tris-HCl (pH 8.5), 200 mM Mg-acetate, 15% polyethylene glycol (PEG) 8000 by the sitting drop vapor diffusion method at 16 °C. This was performed by mixing 0.2 μl protein complex solution (60 μg/μl) with 0.1 μl reservoir solution and equilibrating against 70 μl well solution. The initial crystals were small and were not easily improved by simple changes in pH, chemical concentration, and incubation temperature. To improve crystallization, a 1.5-fold molar excess of purified HPr was supplemented to the purified MtlR-HPr complex and incubated for 30 min at 4 °C until crystallization screening. A bigger crystal was obtained in the same reservoir solution and the condition was further optimized by increasing the incubation temperature up to 25 °C. In the optimized condition, the complex was crystallized without the seeding procedure and fully grew in 2 days.

For the X-ray diffraction experiment, the crystals were dehydrated by adding 0.25 μl of the cryoprotectant solution (the reservoir solution supplemented with 40% [v/v] ethylene glycol) into the crystal-containing drop (0.1–0.2 μl). The crystals were cryo-cooled in a 100 K nitrogen stream. The X-ray diffraction data were collected at Beamline 5C – SB II in the Pohang Accelerator Laboratory (PAL) at a wavelength of 0.98 Å. The diffraction data set was processed by HKL-2000 (HKL Research). The structure was determined by molecular replacement using PHASER in PHENIX^30^ using a vpMtlR monomer of the dimer structure^11^ (PDB 3BRJ) and the H15D mutant structure of HPr^31^ (PDB 1CM3) as search models. Two MtlR-HPr heterodimers  were found in the asymmetric unit: Complex 1 comprises chains A and B and complex 2 comprises chains B and D. Model building and refinement were carried out using COOT^32^ and PHENIX. The Ramachandran plot of the final model showed 94.55% and 5.45% residues in favored and allowed regions, respectively. The coordinates and structure factors have been deposited in the Protein Data Bank (PDB entry 6KCR).

### SEC-MALS

Purified HisMtlR alone (40 μg) and in complex with HPr (60 μg) were analyzed by size exclusion chromatography (SEC) coupled with multi angle light scattering (MALS) using a Superdex^TM^ 200 increase 5/150 GL column (GE Healthcare Life Sciences) on Agilent Technologies 1260 Infinity. The mobile phase (the binding buffer) was applied at a flow rate of 0.2 ml/min. The signals of UV, light scattering and refractive index were respectively monitored by UV/RI detector (Agilent Technologies 1260 Infinity) and miniDAWN TREOS (Wyatt). The data was processed by ASTRA 6.1 (Wyatt). The specific refractive index of BSA (0.1850 ml/g) was used for the determination of the protein concentration.

### Protein thermal stability measurements

Fluorescence labeling of purified HPr was performed following the protocol for N-hydroxysuccinimide (NHS) coupling of the dye NT-647 (NanoTemper Technologies) to lysine residues. Briefly, 100 μl of a 20 mM solution of HPr protein in labeling buffer (20 mM Na-Pi, pH 7.5, 100 mM NaCl and 10% glycerol) was mixed with 100 μl of 60 mM NT-647-NHS fluorophore (NanoTemper Technologies) in labeling buffer and incubated for 30 min at room temperature in the dark. Unbound fluorophores were removed by size-exclusion chromatography in the binding buffer.

The interactions between HPr and MtlR variants were established on a Monolith NT. 115 instrument (NanoTemper Technologies). For this, serial dilutions of MtlR variants were prepared in the binding buffer (20 mM Tris-HCl, pH 7.5, 50 mM NaCl, 2 mM β-mercaptoethanol, 0.05% BSA, 0.05% Tween-20 and 5% glycerol) and mixed 1:1 with a solution of labeled HPr to yield a final volume of 15 μl per dilution. These reaction mixtures were analyzed by microscale thermophoresis as described in Supplementary Fig. 3.

### Calculating the conservation score from multiple sequence alignment

To select non-redundant MtlR sequences, amino acid sequences of MtlRs from 20,000 genomes of the *Enterobacteriaceae* family and 1000 non-*Enterobacteriaceae* genomes were collected from the NCBI database and highly identical sequences (identity >0.7) were filtered using CD-hit^33,34^. From the resulting list of each category, sequences of 1,000 *Enterobacteriaceae* and 21 non-*Enterobacteriaceae* MtlRs were aligned by MUSCLE^35^. After sequence alignment, the conservation score of each amino acid residue was calculated by SCORESCON.

## Supplementary information

Supplementary Information

## Data Availability

All data generated during this study are available from the corresponding authors on reasonable request.

## References

[1] Magasanik B (1961). Catabolite repression. Cold Spring Harb Symp Quant Biol.

[2] Deutscher J, Francke C, Postma PW (2006). How phosphotransferase system-related protein phosphorylation regulates carbohydrate metabolism in bacteria. Microbiol Mol Biol Rev.

[3] Gorke B, Stulke J (2008). Carbon catabolite repression in bacteria: many ways to make the most out of nutrients. Nat Rev Microbiol.

[4] Deutscher J (2014). The bacterial phosphoenolpyruvate:carbohydrate phosphotransferase system: regulation by protein phosphorylation and phosphorylation-dependent protein-protein interactions. Microbiol Mol Biol Rev.

[5] Lee CR, Park YH, Min H, Kim YR, Seok YJ (2019). Determination of protein phosphorylation by polyacrylamide gel electrophoresis. J Microbiol.

[6] Hogema BM (1998). Inducer exclusion in Escherichia coli by non-PTS substrates: the role of the PEP to pyruvate ratio in determining the phosphorylation state of enzyme IIAGlc. Mol Microbiol.

[7] Park YH, Lee BR, Seok YJ, Peterkofsky A (2006). *In vitro* reconstitution of catabolite repression in Escherichia coli. J Biol Chem.

[8] Postma PW, Lengeler JW, Jacobson GR (1993). Phosphoenolpyruvate:carbohydrate phosphotransferase systems of bacteria. Microbiol Rev.

[9] Monod, J. Recherches sur la Croissance des Cultures Bacteriennes. *Thesis, Hermann et Cie, Paris* (1942).

[10] Choe M, Park YH, Lee CR, Kim YR, Seok YJ (2017). The general PTS component HPr determines the preference for glucose over mannitol. Sci Rep.

[11] Tan K (2009). The mannitol operon repressor MtlR belongs to a new class of transcription regulators in bacteria. J Biol Chem.

[12] Figge RM, Ramseier TM, Saier MH (1994). The mannitol repressor (MtlR) of Escherichia coli. J Bacteriol.

[13] Cornilescu G (2002). Solution structure of the phosphoryl transfer complex between the cytoplasmic A domain of the mannitol transporter IIMannitol and HPr of the Escherichia coli phosphotransferase system. J Biol Chem.

[14] Park YH, Um SH, Song S, Seok YJ, Ha NC (2015). Structural basis for the sequestration of the anti-sigma(70) factor Rsd from sigma(70) by the histidine-containing phosphocarrier protein HPr. Acta Crystallogr D Biol Crystallogr.

[15] Kim HM, Park YH, Yoon CK, Seok YJ (2015). Histidine phosphocarrier protein regulates pyruvate kinase A activity in response to glucose in Vibrio vulnificus. Mol Microbiol.

[16] Park YH, Lee CR, Choe M, Seok YJ (2013). HPr antagonizes the anti-sigma70 activity of Rsd in Escherichia coli. Proc Natl Acad Sci USA.

[17] Rothe FM, Bahr T, Stulke J, Rak B, Gorke B (2012). Activation of Escherichia coli antiterminator BglG requires its phosphorylation. Proc Natl Acad Sci USA.

[18] Seok YJ (1997). High affinity binding and allosteric regulation of Escherichia coli glycogen phosphorylase by the histidine phosphocarrier protein, HPr. J Biol Chem.

[19] Blokesch M (2012). Chitin colonization, chitin degradation and chitin-induced natural competence of Vibrio cholerae are subject to catabolite repression. Environ Microbiol.

[20] Davis SJ, Davies EA, Tucknott MG, Jones EY, van der Merwe PA (1998). The role of charged residues mediating low affinity protein-protein recognition at the cell surface by CD2. Proc Natl Acad Sci USA.

[21] Sheinerman FB, Norel R, Honig B (2000). Electrostatic aspects of protein-protein interactions. Curr Opin Struct Biol.

[22] Zhao N, Pang B, Shyu CR, Korkin D (2011). Charged residues at protein interaction interfaces: unexpected conservation and orchestrated divergence. Protein Sci.

[23] Zhao H, Brown PH, Schuck P (2011). On the distribution of protein refractive index increments. Biophys J.

[24] Garrett DS, Seok YJ, Peterkofsky A, Gronenborn AM, Clore GM (1999). Solution structure of the 40,000 Mr phosphoryl transfer complex between the N-terminal domain of enzyme I and HPr. Nat Struct Biol.

[25] Ha JH (2018). Evidence of link between quorum sensing and sugar metabolism in Escherichia coli revealed via cocrystal structures of LsrK and HPr. Sci Adv.

[26] Jung YS, Cai M, Clore GM (2012). Solution structure of the IIAChitobiose-HPr complex of the N,N′-diacetylchitobiose branch of the Escherichia coli phosphotransferase system. J Biol Chem.

[27] Wang G (2000). Solution structure of the phosphoryl transfer complex between the signal transducing proteins HPr and IIA(glucose) of the Escherichia coli phosphoenolpyruvate:sugar phosphotransferase system. EMBO J.

[28] Williams DC, Cai M, Suh JY, Peterkofsky A, Clore GM (2005). Solution NMR structure of the 48-kDa IIAMannose-HPr complex of the Escherichia coli mannose phosphotransferase system. J Biol Chem.

[29] Datsenko KA, Wanner BL (2000). One-step inactivation of chromosomal genes in Escherichia coli K-12 using PCR products. Proc Natl Acad Sci USA.

[30] Adams PD (2010). PHENIX: a comprehensive Python-based system for macromolecular structure solution. Acta Crystallogr D Biol Crystallogr.

[31] Napper S, Delbaere LT, Waygood EB (1999). The aspartyl replacement of the active site histidine in histidine-containing protein, HPr, of the Escherichia coli Phosphoenolpyruvate:Sugar phosphotransferase system can accept and donate a phosphoryl group. Spontaneous dephosphorylation of acyl-phosphate autocatalyzes an internal cyclization. J Biol Chem.

[32] Emsley P, Lohkamp B, Scott WG, Cowtan K (2010). Features and development of Coot. Acta Crystallogr D Biol Crystallogr.

[33] Fu L, Niu B, Zhu Z, Wu S, Li W (2012). CD-HIT: accelerated for clustering the next-generation sequencing data. Bioinformatics.

[34] Li W, Godzik A (2006). Cd-hit: a fast program for clustering and comparing large sets of protein or nucleotide sequences. Bioinformatics.

[35] Edgar RC (2004). MUSCLE: multiple sequence alignment with high accuracy and high throughput. Nucleic Acids Res.

[36] Eisenberg D, Schwarz E, Komaromy M, Wall R (1984). Analysis of membrane and surface protein sequences with the hydrophobic moment plot. J Mol Biol.

