# Supplementary material for: Structural insight into glucose repression of the mannitol operon
Source: Sci Rep. 2019 Sep 26;9:13930. doi: 10.1038/s41598-019-50249-2 (PMC6763467; doi:10.1038/s41598-019-50249-2)
Supplement: Supplementary file 1 — Supplementary Information [file 41598_2019_50249_MOESM1_ESM.pdf]

Structural insight into glucose repression of the mannitol operon.

Mangyu Choe[1†], Huitae Min[1†], Young-Ha Park[1], Yeon-Ran Kim[1], Jae-Sung Woo[2*] & Yeong-Jae Seok[1*]

[1]School of Biological Sciences and Institute of Microbiology, Seoul National University, Seoul 00826, Korea.

[2]Department of Life Sciences, Korea University, 145 Anam-ro, Seongbuk-gu, Seoul 02841, Republic of Korea

[†]Mangyu Choe and Huitae Min equally contributed to this work.

[*]Correspondence and requests for materials should be addressed to J.S.W. (e-mail: jaesungwoo@korea.ac.kr) or Y.J.S. (e-mail: yjseok@snu.ac.kr).

Supplementary Informations

Supplementary Figures 1-8

Supplementary Table 1-2

Supplementary references

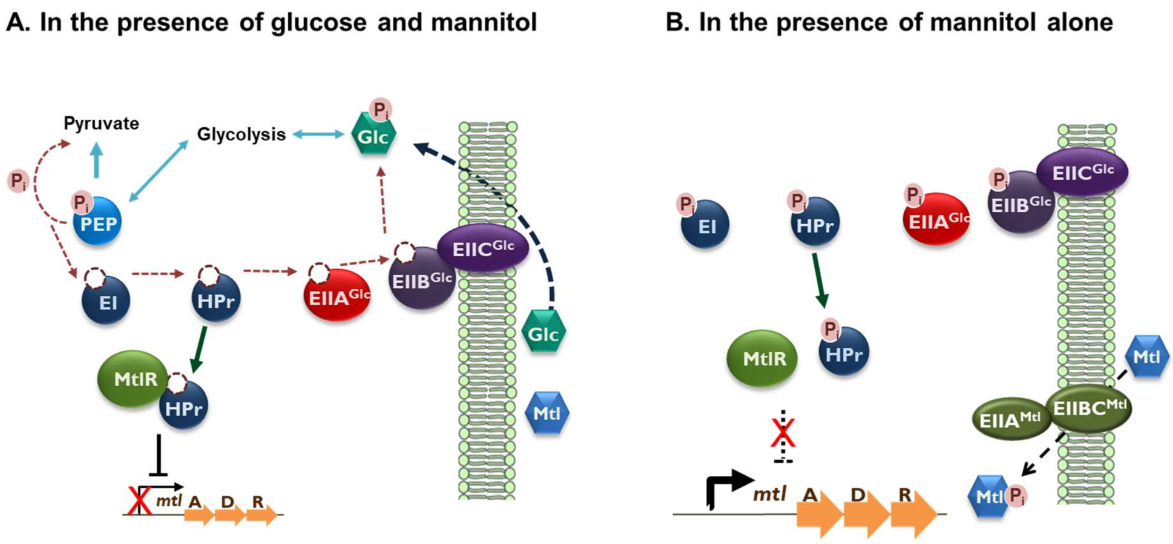

Supplementary Figure 1. A schematic view of the current model for glucose repression of the *mtl* operon in *E. coli*.

In the presence of glucose, the phosphoryl group of phosphoenolpyruvate (PEP) is sequentially transferred through EI, HPr and EII^{Glc} to glucose, and therefore HPr and $EIIA^{Glc}$ are predominantly dephosphorylated. Dephospho-HPr interacts with MtlR, a transcriptional repressor of the *mtl* operon, to augment its repressor activity. In the absence of glucose, however, phosphorylation of HPr increases. Since phosphorylation of HPr hinders the formation of the HPr-MtlR complex, the *mtl* operon can be induced.

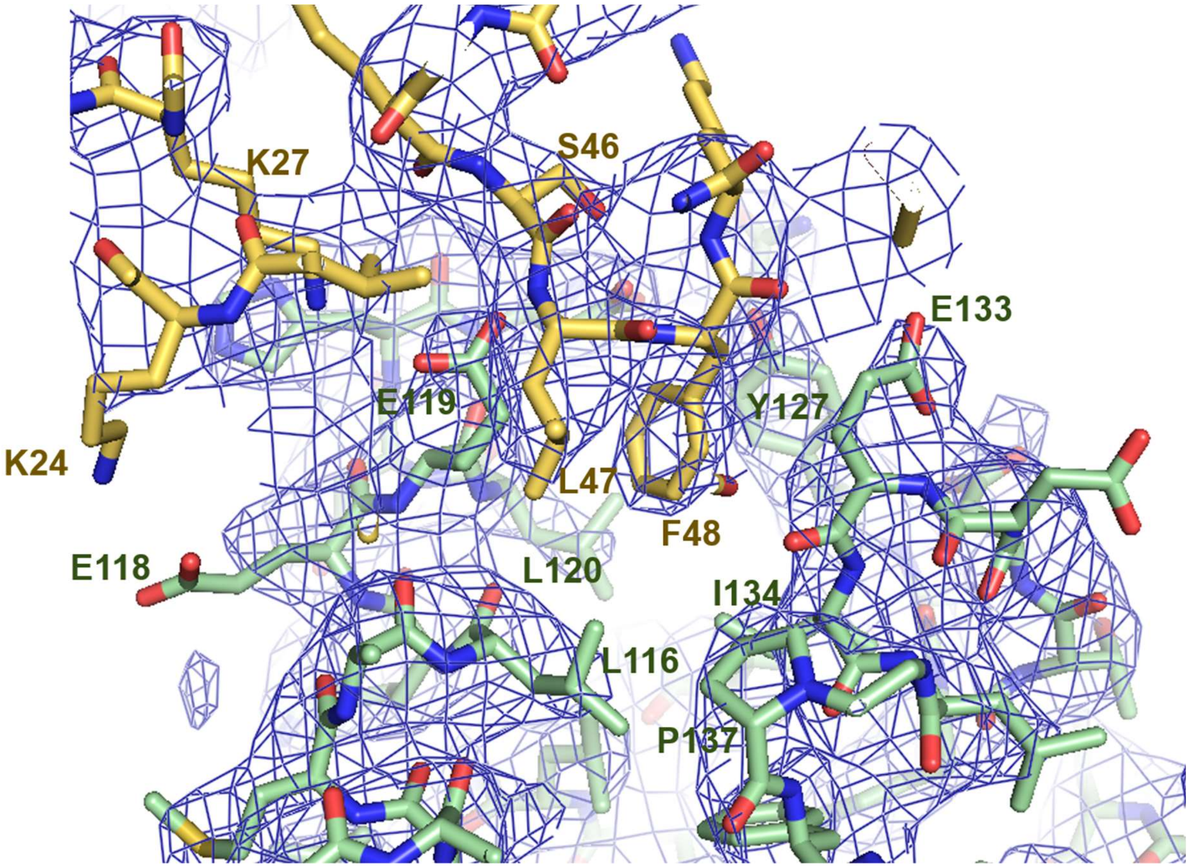

Supplementary Figure 2. Close-up view of the binding interface between MtlR and HPr. MtlR, HPr and electron density are shown in green (stick), yellow (stick) and blue (mesh), respectively. The residues participating in the intermolecular interaction are labeled.

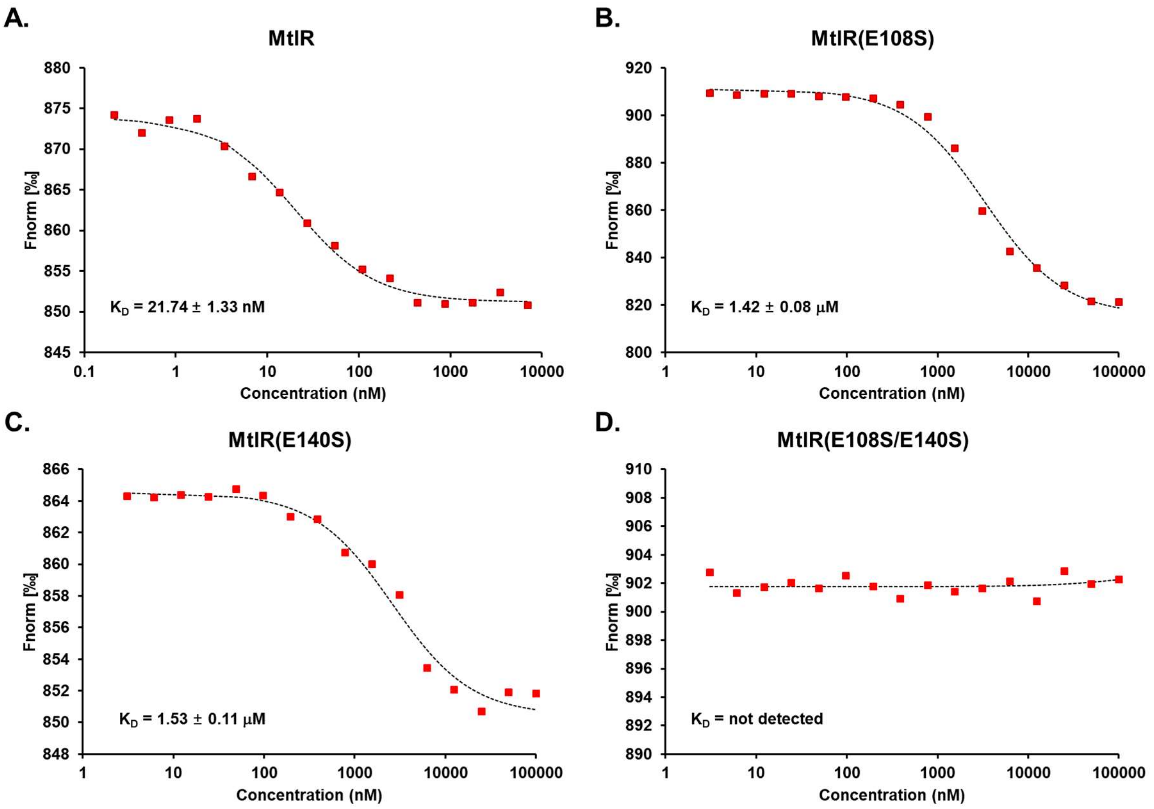

Supplementary Figure 3. Measurement of the dissociation constant (K_D) between MtlR variants and HPr.

Each purified MtlR variant was serially diluted and mixed with fluorescence-labeled HPr. The reaction mixture was loaded into a standard treated capillary (NanoTemper Technologies) and subsequently analyzed by microscale thermophoresis (MST) at 20% MST power and a light-emitting diode (LED) intensity of 10%. Analysis of the interaction by thermophoresis was performed with a laser on for 30 s. All experiments were repeated three times and representative data are shown here. The K_D values and standard deviations were determined using MO Affinity Analysis software version 2.2.7.

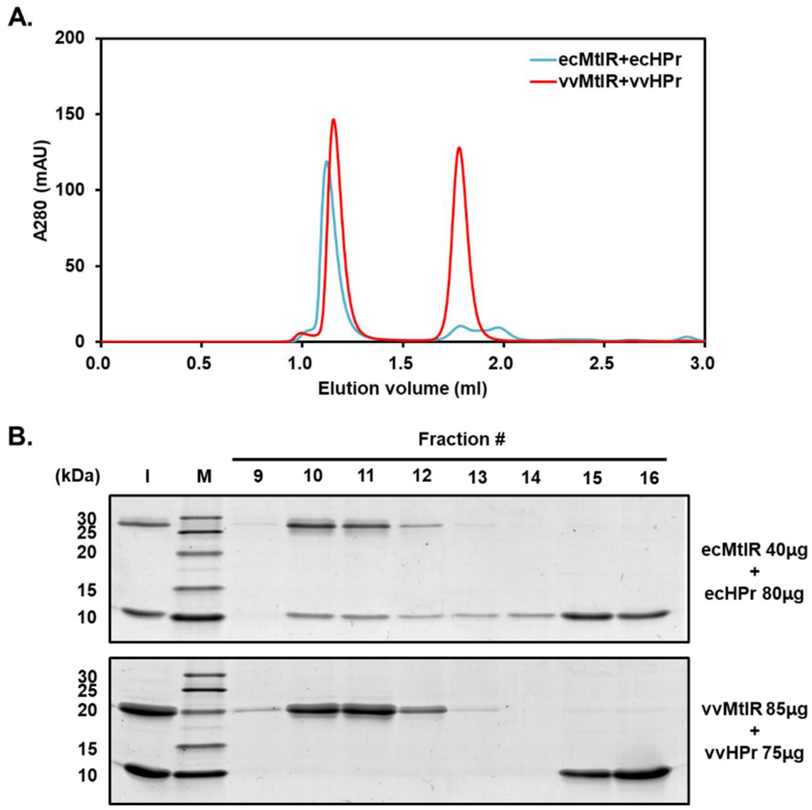

Supplementary Figure 4. Gel filtration chromatography of the MtlR-HPr complex of *E. coli* and *V. vulnificus*.

Gel filtration chromatography was performed on a Superdex 75 increase 5/150 GL column (GE Healthcare Life Sciences) equilibrated with the running buffer containing 20 mM Tris-HCl (pH 7.5), 50 mM NaCl, 2 mM β-mercaptoethanol and 5% glycerol at a flow rate of 0.2 ml/min using an Agilent 1260 infinity Binary LC system. (A) A protein mixture containing *E. coli* MtlR (40 μg) and *E. coli* HPr (80 μg) was prepared in 50 μl of the running buffer (blue line) and one containing *V. vulnificus* MtlR (85 μg) and *V. vulnificus* HPr (75 μg) in 20 μl of the running buffer (red line). Both mixtures were incubated for 10 min on ice and then injected into the column, and their absorbance profiles at 280 nm are shown. It should be noted *E. coli* HPr exhibits a significantly lower absorbance at 280 nm than *V. vulnificus* HPr, since the former has no tryptophan, tyrosine or cysteine whereas the latter has a tyrosine residue. (B) Fractions (120 μl each) from the gel filtration column were collected, and 10 μl of each fraction was analyzed by 4-20% SDS-PAGE followed by staining with Coomassie brilliant blue R-250. Lane M indicates the PageRuler^{TM} Unstained Protein Ladder (Thermo Scientific), and the molecular masses (in kDa) of some standards are presented on the left. "I" indicates an aliquot of the injected mixture.

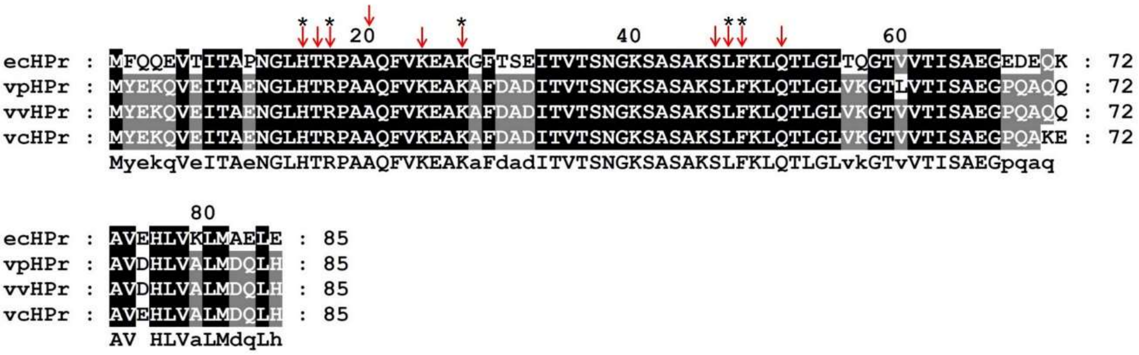

Supplementary Figure 5. Amino acid sequence alignment of HPr among *E. coli* and *Vibrio* species using ClustalX2.

Conserved residues are highlighted in black (100%) and gray (75%). The residues identified to be involved in the interaction with MtlR in this study are indicated by red arrows and the five residues tested for their involvement in the interaction with MtlR in a previous study[1] are indicated by asterisks. ec, *E. coli*; vp, *V. parahaemolyticus*; vv, *V. vulnificus*; vc, *V. cholerae*.

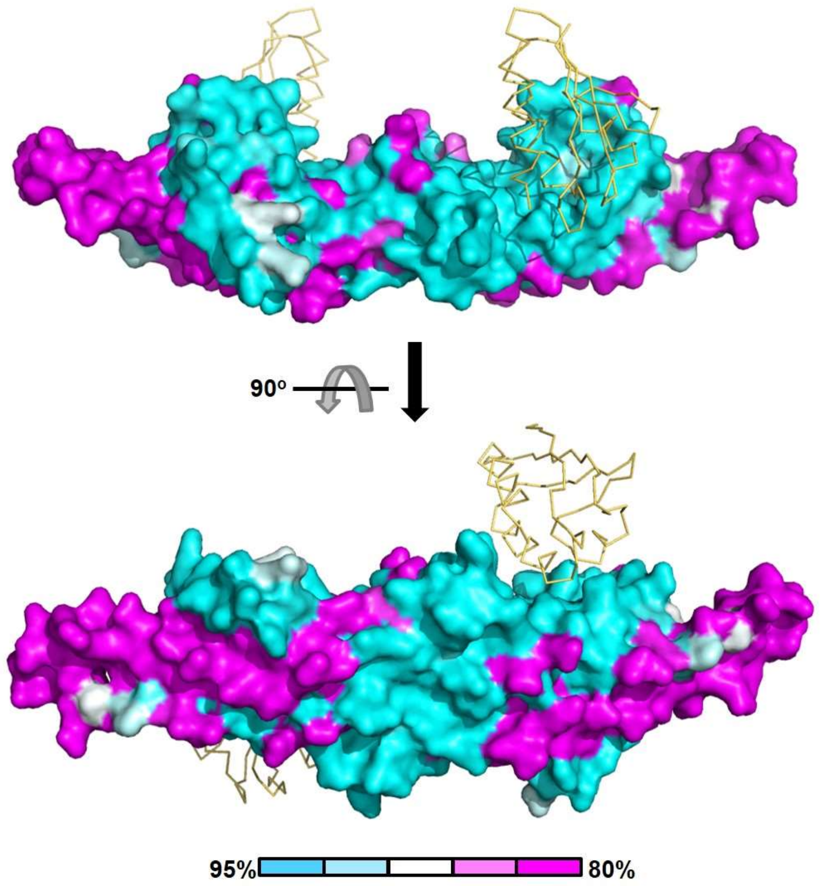

residue	conservation rate (%)
Tyr107	100
Glu108	100
Glu111	100
Leu112	99.7
Ala115	99.8
Leu116	90.7
Glu118	100
Glu119	98.1
Leu120	98.1
His122	95.8
Asp123	99.7
Tyr127	100
Glu133	99.2
Ile134	95.8
Glu140	99.9

Supplementary Figure 6. The HPr-binding site is highly conserved in *Enterobacteriaceae* MtlRs.

The conservation rate of each residue of MtlR was calculated by multiple sequence alignment. Amino acid sequences of MtlRs from 1000 *Enterobacteriaceae* genomes were aligned and the residue conservation rates are color-coded onto the surface of *E. coli* MtlR. The color bar indicates the conservation rate.

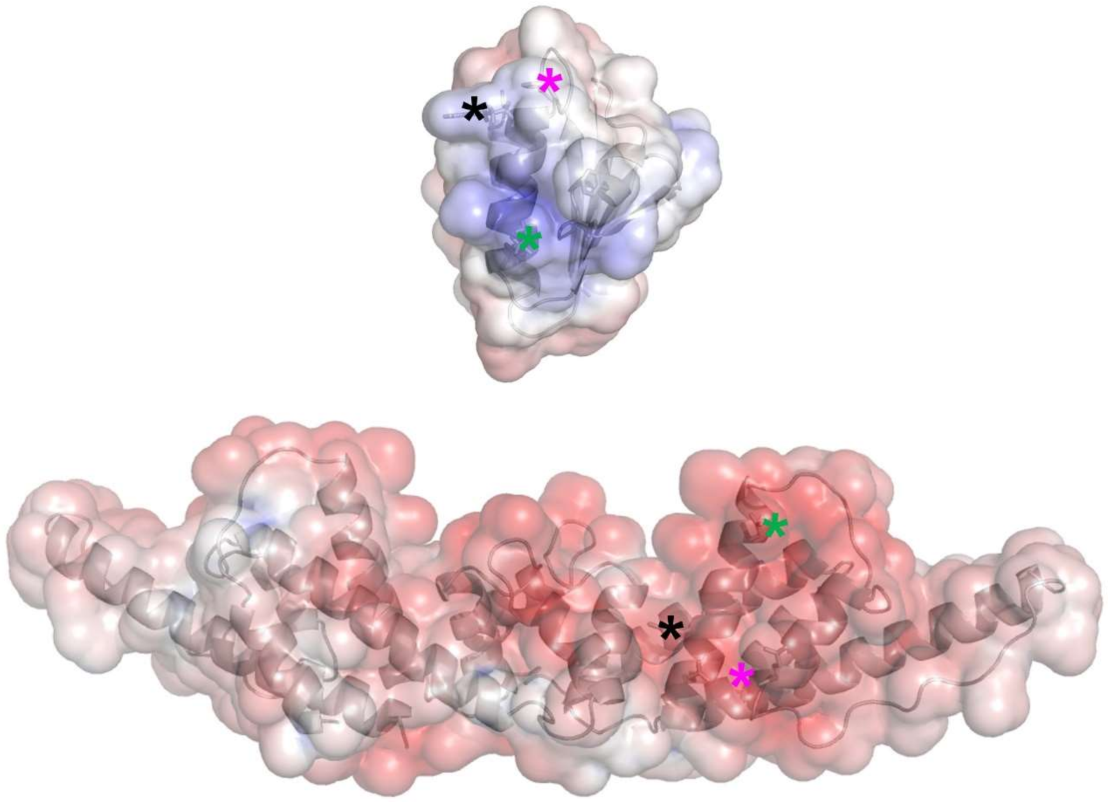

Supplementary Figure 7. Three electrostatic interaction pairs in the binding interface of MtlR and HPr are important for their binding affinity and specificity.

Three electrostatic interaction pairs are marked with asterisks in magenta (H15 of HPr with E108 and E140 of MtlR), black (R17 of HPr with E111 of MtlR) and green (K27E of HPr with E119 of MtlR). Surface electrostatic potential is generated as described in the legend to Figure 4, and represented as a transparent surface.

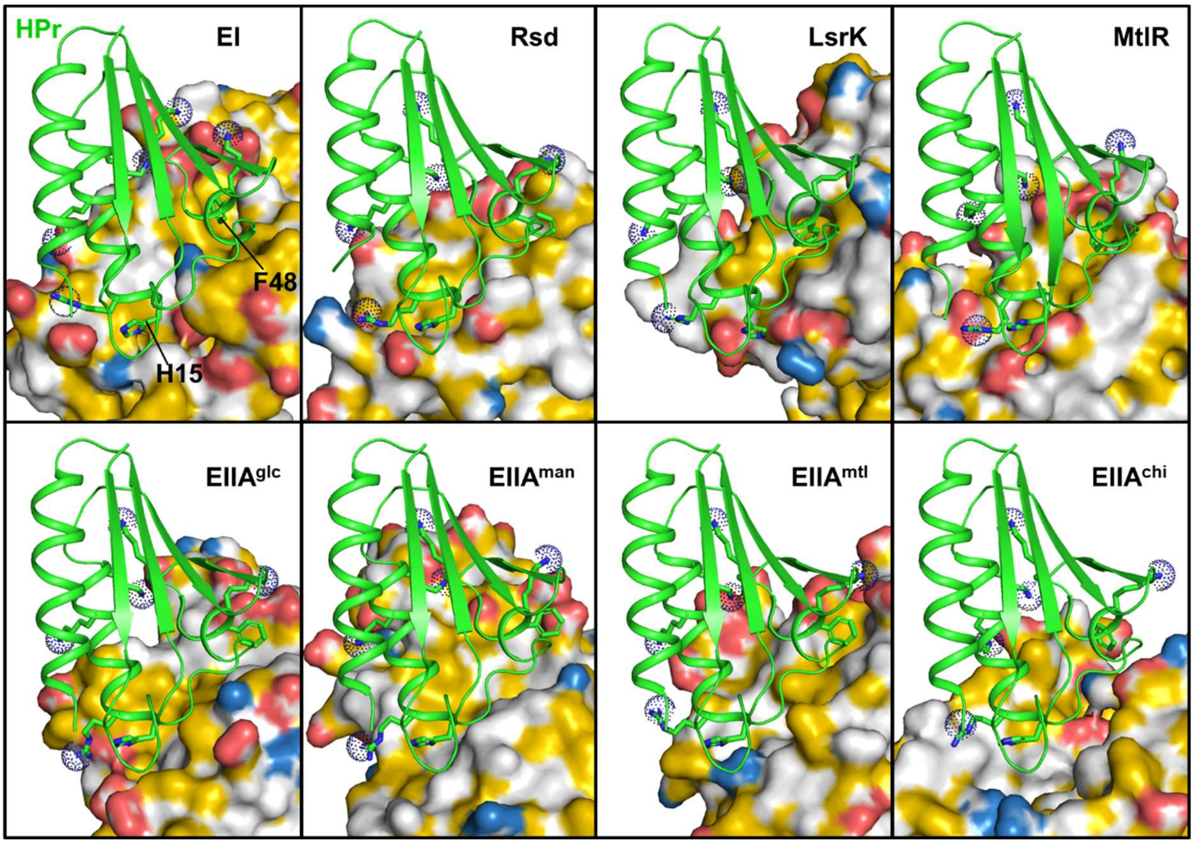

Supplementary Figure 8. HPr recognition mechanisms of various HPr-binding proteins. HPr-binding proteins are shown as surface presentations with YRB highlighting[2]. Hydrophobic atoms, negatively charged atoms, and positively charged atoms are colored yellow, red, and blue, respectively. HPr molecules (green) are at the same orientation and shown as ribbon drawings. Phe48 and His15 of HPr are in sticks and labeled. Four lysine and one arginine residues on the basic surface of HPr are shown as sticks, and positively charged nitrogen atoms in the residues are highlighted by dots. PDB accession codes for coordinates are 3EZE (EI), 4XWJ (RsdI), 5YA1 (LsrK), 1GGR ($EIIA^{Glc}$), 1VRC ($EIIA^{Man}$), 1J6T ($EIIA^{Mtl}$), and 2LRK ($EIIA^{Chi}$).

Supplementary Table 1. Data collection and refinement statistics.

	MtlR-Hpr
**Data collection**	
Space group	*I222*
Cell dimensions	
a, *b*, *c* (Å)	97.4, 122.3, 147.5
α, β, γ (°)	90, 90, 90
Resolution (Å)	30 – 3.5
R_{sym} or R_{merge} (%)	7.2 (73.0)*
$I / \sigma I$	22.3 (1.7)
Completeness (%)	99.8 (100)
Redundancy	6.4 (6.3)
**Refinement**	
Resolution (Å)	30 – 3.5
No. reflections	11374
R_{work} / R_{free}	24.0 / 26.0
No. atoms	
Protein	3908
B-factors	
Protein	138.2
R.m.s deviations	
Bond lengths (Å)	0.002
Bond angles (°)	0.538
Ramachandran plot (%)	
Favored	94.55
Allowed	5.45

*Highest resolution shell is shown in parenthesis.

Supplementary Table 2. Bacterial strains and plasmids used in this study.

Strains or plasmid	Genotype or phenotype	Source or Reference
***E. coli* strains**		
MG1655	Wild-type *E. coli* K-12	[3]
MG1655 mtlR(E108/140S)	MG1655 *mtlR*::mtlR (E108S/E140S)	This study
GI698	F^- λ^- *lacI*^{q} *lacPL8 ampC*::P_{trp} cI	[4]
GI698Δ*pts*	GI698 *ptsHIcrr*::Km^r	[5]
ER2566	F- λ- f*huA2* [*lon*] *ompT lacZ::T7 gene 1 gal sulA11* Δ(*mcrC-mrr*)*114::IS10 R*(*mcr-73::miniTn10-TetS*)*2 R*(*zgb-210::Tn10*)(*TetS*) *endA1* [*dcm*]	New England Biolabs
***V. vulnificus* strains**		
CMCP6	Clinical isolate	[6]
**Plasmid**		
pRE1	Expression vector under control of λP_L promoter, Amp^r	[7]
pSP100	pRE1-based expression vector for HPr	[8]
pET43.1a	Cloning vector; Amp^r	Novagen
pET-MtlR	*E. coli mtlR* ORF cloned between NdeI and XhoI sites of pET43.1a	[1]
pET-HisMtlR	His_6 tag added to the N-terminus of MtlR in pET-MtlR	This study
pET-HisMtlR(E108S)	Glu108 of MtlR in pET-HisMtlR was mutated to Ser	This study
pET-HisMtlR(E140S)	Glu140 of MtlR in pET-HisMtlR was mutated to Ser	This study
pET-HisMtlR(L112A)	Lys112 of MtlR in pET-HisMtlR was mutated to Ala	This study
pET-HisMtlR(A115W)	Ala115 of MtlR in pET-HisMtlR was mutated to Trp	This study
pET-HisMtlR(E108S/E140S)	Glu108 and Glu140 of MtlR in pET-HisMtlR were mutated to Ser	This study
pETDeut	Cloning vector; Amp^r	Novagen
pETDeut-vvHisMtlR	*V. vulnificus mtlR* ORF cloned between NdeI and XhoI sites of pETDeut	This study
pRK415	Broad host range vector, IncP *ori*, *oriT* of RK2; Tc^r	[9]
pKD46	Red recombinase expression plasmid under the control of arabinose inducible promoter	[10]

Supplementary References

1 Choe, M., Park, Y. H., Lee, C. R., Kim, Y. R. & Seok, Y. J. The general PTS component HPr determines the preference for glucose over mannitol. *Sci Rep* **7**, 43431, doi:10.1038/srep43431 (2017).

2 Hagemans, D., van Belzen, I. A., Moran Luengo, T. & Rudiger, S. G. A script to highlight hydrophobicity and charge on protein surfaces. *Front Mol Biosci* **2**, 56, doi:10.3389/fmolb.2015.00056 (2015).

3 Blattner, F. R. *et al.* The complete genome sequence of Escherichia coli K-12. *Science* **277**, 1453-1462 (1997).

4 LaVallie, E. R. *et al.* A thioredoxin gene fusion expression system that circumvents inclusion body formation in the E. coli cytoplasm. *Biotechnology (N Y)* **11**, 187-193 (1993).

5 Nosworthy, N. J. *et al.* Phosphorylation destabilizes the amino-terminal domain of enzyme I of the Escherichia coli phosphoenolpyruvate:sugar phosphotransferase system. *Biochemistry* **37**, 6718-6726, doi:10.1021/bi980126x (1998).

6 Kim, Y. R. *et al.* Characterization and pathogenic significance of Vibrio vulnificus antigens preferentially expressed in septicemic patients. *Infect Immun* **71**, 5461-5471 (2003).

7 Reddy, P., Peterkofsky, A. & McKenney, K. Hyperexpression and purification of Escherichia coli adenylate cyclase using a vector designed for expression of lethal gene products. *Nucleic Acids Res* **17**, 10473-10488 (1989).

8 Garrett, D. S., Seok, Y. J., Peterkofsky, A., Clore, G. M. & Gronenborn, A. M. Identification by NMR of the binding surface for the histidine-containing phosphocarrier protein HPr on the N-terminal domain of enzyme I of the Escherichia coli phosphotransferase system. *Biochemistry* **36**, 4393-4398, doi:10.1021/bi970221q (1997).

9 Keen, N. T., Tamaki, S., Kobayashi, D. & Trollinger, D. Improved broad-host-range plasmids for DNA cloning in gram-negative bacteria. *Gene* **70**, 191-197 (1988).

10 Datsenko, K. A. & Wanner, B. L. One-step inactivation of chromosomal genes in Escherichia coli K-12 using PCR products. *Proc Natl Acad Sci U S A* **97**, 6640-6645, doi:10.1073/pnas.120163297 (2000).
